# A Novel Biosynthetic Strategy for Ginsenoside Ro: Construction of a Metabolically Engineered *Saccharomyces cerevisiae* Strain Using a Newly Identified UGAT Gene from *Panax ginseng* as the Key Enzyme Gene and Optimization of Fermentation Conditions

**DOI:** 10.3390/ijms252011331

**Published:** 2024-10-21

**Authors:** Xiaochen Yu, Jinghui Yu, Dinghui Wang, Sizhang Liu, Kangyu Wang, Mingzhu Zhao, Ping Chen, Yanfang Wang, Yi Wang, Meiping Zhang

**Affiliations:** 1 College of Life Science, Jilin Agricultural University, Changchun 130118, China; yu-xiaochen@foxmail.com (X.Y.); jinghui-yu@foxmail.com (J.Y.); dinghui-wang@foxmail.com (D.W.); lsz512411412@163.com (S.L.); kangyu.wang@jlau.edu.cn (K.W.); mingzhuzhao@jlau.edu.cn (M.Z.); chenping201407@126.com (P.C.); 2 Jilin Engineering Research Center Ginseng Genetic Resources Development and Utilization, Jilin Agricultural University, Changchun 130118, China; 3 College of Chinese Medicinal Materials, Jilin Agricultural University, Changchun 130118, China; yfwang2014@163.com

**Keywords:** *Panax ginseng*, ginsenoside, biosynthesis, UDP-glucuronosyltransferase

## Abstract

Ginsenoside Ro, as one of the few oleanane-type ginsenosides, is well known for its unique molecular structure and biological activities. Currently, research on the biosynthesis of ginsenoside Ro is still in its early stages. Therefore, the establishment of a new ginsenoside Ro cell factory is of great significance for the in-depth development and utilization of genes related to ginsenoside Ro synthesis, as well as for the exploration of pathways to obtain ginsenoside Ro. In this study, we cloned endogenous constitutive promoters, terminators, and other genetic elements from *S. cerevisiae* BY4741. These elements were then sequentially assembled with the uridine diphosphate glucuronic acid transferase gene identified in our previously study (*PgUGAT252645*) and several other reported key enzyme genes, to construct DNA fragments used for integration into the genome of *S. cerevisiae* BY4741. By sequentially transferring these DNA fragments into chemically competent cells of engineering strains and conducting screening and target product detection, we successfully constructed an engineered *S. cerevisiae* strain (BY-Ro) for ginsenoside Ro biosynthesis using *S. cerevisiae* BY4741 as the host cell. Strain BY-Ro produced 253.32 μg/L of ginsenoside Ro under optimal fermentation conditions. According to subsequent measurements and calculations, this equates to 0.033 mg/g DCW, corresponding to approximately 31% of the ginsenoside Ro content found in plant samples. This study not only included a deeper investigation into the function of *PgUGAT252645* but also provides a novel engineering platform for ginsenoside Ro biosynthesis.

## 1. Introduction

Ginsenosides are a class of secondary metabolites from plants of the *Panax* genus belonging to the triterpenoid class [1], generally with high medicinal value. To date, approximately 180 ginsenosides have been isolated and identified from 17 species of the *Panax* genus [2,3,4]. The differences in the molecular structures of different monomeric ginsenosides lead to general differences in their pharmacological activities. Among them, ginsenoside Ro is a highly specific ginsenoside. Ginsenoside Ro is one of the few reported oleanane-type ginsenosides to date [5]; its triterpene molecular skeleton is a pentacyclic triterpene compound, namely oleanolic acid [6]. In addition, among all the monomeric ginsenosides, ginsenoside Ro is one of the few ginsenosides known to contain a glucuronic acid group in its molecular structure.

Currently, the main method for obtaining ginsenosides is direct extraction from plant tissue. This production method is often limited by the efficiency of the extraction process, which, in turn, leads to a yield that struggles to meet market demand [7]. Although efficient systems for obtaining ginsenosides have been successfully established through biotechnological means such as cell engineering [8], metabolic engineering [9], and genetic engineering [10], these research results still face various limitations in practical applications, including with respect to cost and safety.

With the continuous development of synthetic biology, more and more active ingredients of medicinal plants have achieved heterologous biosynthesis and are expected to replace traditional planting and extraction methods and become a new way to obtain medicinal ingredients. Since the first study on the synthesis of dammarenediol-II and protopanaxadiol using *S. cerevisiae* was reported [11], research on the synthetic biology of ginsenosides using *E. coli* [12] and tobacco [13,14,15] as host organisms has also been carried out. Since the cell structure of *S. cerevisiae* is similar to that of plant cells, it can provide a suitable environment for cytochrome P450 and glycosyltransferase in the synthesis pathway of ginsenosides [16], and the inherent MVA pathway can provide sufficient precursor substrates [17]. In addition, the higher biosafety and higher tolerance to enzyme proteins encoded by genes from other species make provide advantages in the application of ginsenoside biosynthesis [18].

At present, exogenous key enzyme-encoding genes from species such as *P. notoginseng* [19], *G. uralensis* [20], *A. thaliana* [21], *T. subterraneum* [22], and *E. coli* [23] have been successively applied to the construction of the engineered *S. cerevisiae* strains of ginsenoside biosynthesis. Optimization strategies such as the development of ginsenoside synthesis compartments in engineered yeast strains [24] and modularization of ginsenoside synthesis pathways [25,26] have also been proposed. In addition, studies have shown that optimizing fermentation conditions including culture medium elements can effectively increase the yield of ginsenosides in engineered strains [27,28].

Although current synthetic biology research targeting ginsenoside Ro has made some progress [22,23], the number of known key genes involved in the biosynthesis of ginsenoside Ro is still limited, and the diversity of synthetic pathway construction schemes is insufficient. In the synthesis pathway of ginsenoside Ro, glucuronidation modification of the hydroxyl group at the C3 position of oleanolic acid is the first step in the glycosylation process. In a previous study, we identified uridine diphosphate glucuronic acid transferase gene *PgUGAT252645* [29], which catalyzes this step in *P. ginseng*. In this study, we used *PgUGAT252645* as one of the key enzyme gene elements to construct a new engineered *S. cerevisiae* strain for the biosynthesis of ginsenoside Ro. By optimizing fermentation conditions, we further improved the yield of ginsenoside Ro in the engineered *S. cerevisiae* strain.

## 2. Results

### 2.1. Construction of the Engineered S. cerevisiae Strain

By performing agarose gel electrophoresis and DNA sequencing, it was confirmed that all gene elements of the assembled key enzyme gene expression cassettes and genomic integration fragments were correctly connected in the order designed in the experiment. Competent *S. cerevisiae* cells were then transformed using the described methods, constructing and screening (Figure S1) the engineered *S. cerevisiae* strains with the genotypes shown in Table 1.

**Figure 1:**
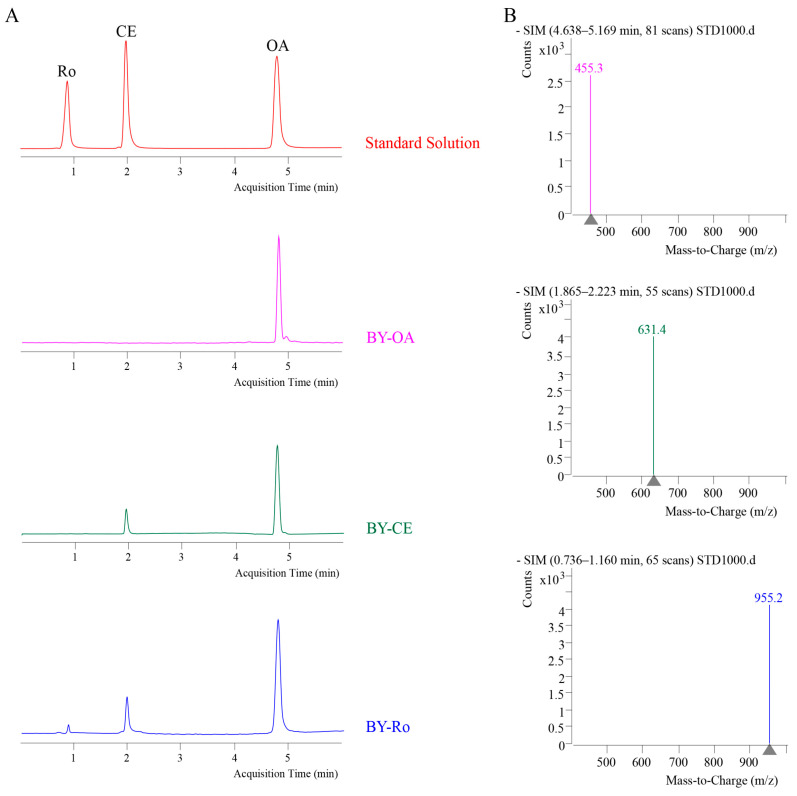
Liquid chromatography–mass spectrometry (LC-MS) detection of target products. (**A**) Liquid chromatographic analysis of the standard and extract samples. The red curve represents the standard solution containing oleanolic acid (OA), calenduloside E (CE), and ginsenoside Ro (OA). The pink, green, and blue curves represent the extract samples from the cultures of strains BY-OA, BY-CE, and BY-Ro, respectively. (**B**) Mass spectrometric detection of the standards.

The results of liquid chromatography–mass spectrometric (LC-MS) detection of target products for each strain during the construction process are shown in Figure 1. The retention times of oleanolic acid (OA), calenduloside E (CE), and ginsenoside Ro (Ro) in the standard solution correspond to the chromatographic peaks detected in the extract solutions of different strain cultures. Mass spectrometry results showed ions with the same mass as the target products at the corresponding times, indicating that the extract solutions of each strain culture contained the respective target products. This confirms that all gene fragments were successfully integrated into the genome and correctly performed their catalytic functions. Based on the sample retention peak areas, the yields of oleanolic acid in strain BY-OA, calenduloside E in strain BY-CE, and ginsenoside Ro in strain BY-Ro were 56.31 mg/L, 3.8 mg/L, and 155 μg/L, respectively.

### 2.2. Optimization of Fermentation Conditions: Single-Factor Experiment

**Figure 2:**
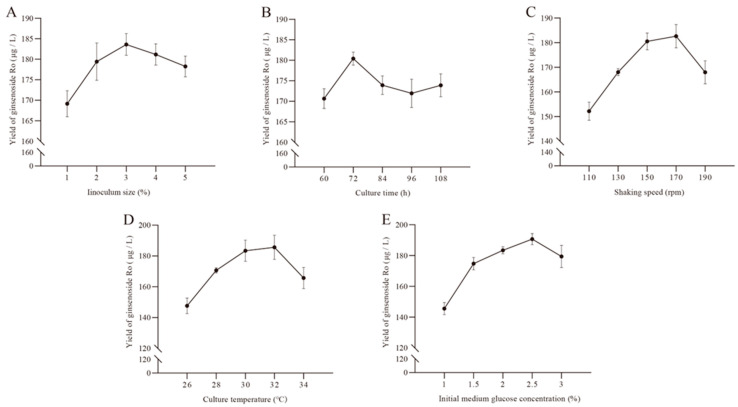
The effect of different cultivation conditions on the yield of ginsenoside Ro in strain BY-Ro. (**A**) Inoculum size. (**B**) Culture time. (**C**) Shaking speed. (**D**) Culture temperature. (**E**) Initial medium glucose concentration.

As shown in Figure 2A, when the inoculum size is 1–3%, the yield of ginsenoside Ro in strain BY-Ro increases with the inoculum size and reaches a maximum of 183.60 μg/L at an inoculum size of 3%. When the inoculum size is greater than 3%, the yield of ginsenoside Ro decreases with increasing inoculum size. Therefore, an inoculum size range of 2–4% was selected for subsequent experiments.

As shown in Figure 2B, the yield of ginsenoside Ro in strain BY-Ro reaches a maximum of 180.39 ng/L at a culture time of 72 h. When the culture time is greater than 72 h, the yield of ginsenoside Ro significantly decreases. Therefore, a culture time range of 60 h–84 h was selected for subsequent experiments.

As shown in Figure 2C, the yield of ginsenoside Ro in strain BY-Ro increases with shaking speed in the range of 110–170 rpm, reaching a maximum of 182.61 μg/L at 170 rpm. When the shaking speed is greater than 170 rpm, the yield of ginsenoside Ro significantly decreases. Therefore, a shaking speed range of 150–190 rpm was selected for subsequent experiments.

As shown in Figure 2D, the yield of ginsenoside Ro in strain BY-Ro reaches a maximum of 185.63 ng/L at a culture temperature at 32 °C. When the culture temperature is higher than 32 °C, the yield of ginsenoside Ro significantly decreases. Therefore, a culture temperature range of 30–34 °C was selected for subsequent experiments.

As shown in Figure 2E, the yield of ginsenoside Ro in strain BY-Ro increases with an initial medium glucose concentration in the range of 1–2.5%, reaching a maximum of 190.70 μg/L at 2.5%. When the initial medium glucose concentration is higher than 2.5%, the yield of ginsenoside Ro decreases. Therefore, an initial medium glucose concentration range of 2–3% in the medium was selected for subsequent experiments.

### 2.3. Optimization of Fermentation Conditions: Plackett–Burman Design

The design of relevant factors and levels in the Plackett–Burman design based on the results of the single-factor experiments is shown in Table 2. Using ginsenoside Ro yield as the response value, the design scheme and experimental data for single-factor significance screening are shown in Table 3. The results of the statistical analysis of the experimental data are shown in Table 4. The analysis results show that the established model has a *p* value of 0.0001 < 0.01, an F value of 104.22 > 1, R^2^ = 0.9886, and R^2^_adj_ = 0.9791, indicating that the model can effectively explain the experimental results and provide guidance for further optimization. Additionally, the multiple linear regression equation between the factors and the response value is expressed as follows:

Y = 188.21 − 0.36A + 1.29B + 3.25C − 28.14D + 10.37E

The results indicate that shaking speed, culture time, and initial medium glucose concentration have positive effects, while inoculum size and culture temperature have negative effects. The order of factors affecting ginsenoside Ro yield is culture temperature > initial medium glucose concentration > shaking speed > culture time > inoculum size. Among them, culture temperature, initial medium glucose concentration, and shaking speed have significant impacts on ginsenoside Ro yield. Therefore, these factors were selected for subsequent response surface optimization design experiments and analysis.

### 2.4. Optimization of Fermentation Conditions: Box–Behnken Design

The relevant factors and levels for the Box-Behnken design based on the analysis of the Plackett–Burman design and experimental results are shown in Table 5. We selected culture temperature, initial medium glucose concentration, and shaking speed, which have significant impacts on ginsenoside Ro yield, as the factors for further optimization. A three-factor, three-level experimental design was implemented. The Box–Behnken design scheme and experimental data obtained using ginsenoside Ro yield as the response value are shown in Table 6. The results of statistical analysis of the experimental data are shown in Table 7. The analysis results show that the established model has a *p* value of 0.0001 < 0.01, an F value of 173.03 > 1, R^2^ = 0.9955, and R^2^adj = 0.9898, indicating that the model can provide adequate guidance for the optimization of fermentation conditions. Additionally, the multiple quadratic regression equation between the factors and the response value is expressed as follows:

Y = 255.98 + 13.24A + 7.09B − 15.73C − 3.98AB + 4.33AC − 6.01BC − 31.59A^2^ − 15.24B^2^ − 29.43C^2^

The results indicate that the initial medium glucose concentration, shaking speed, and culture temperature have a highly significant impact on ginsenoside Ro yield (*p* ≤ 0.01), and the interaction between shaking speed and culture temperature also has a highly significant impact on ginsenoside Ro yield (*p* ≤ 0.01).

**Figure 3:**
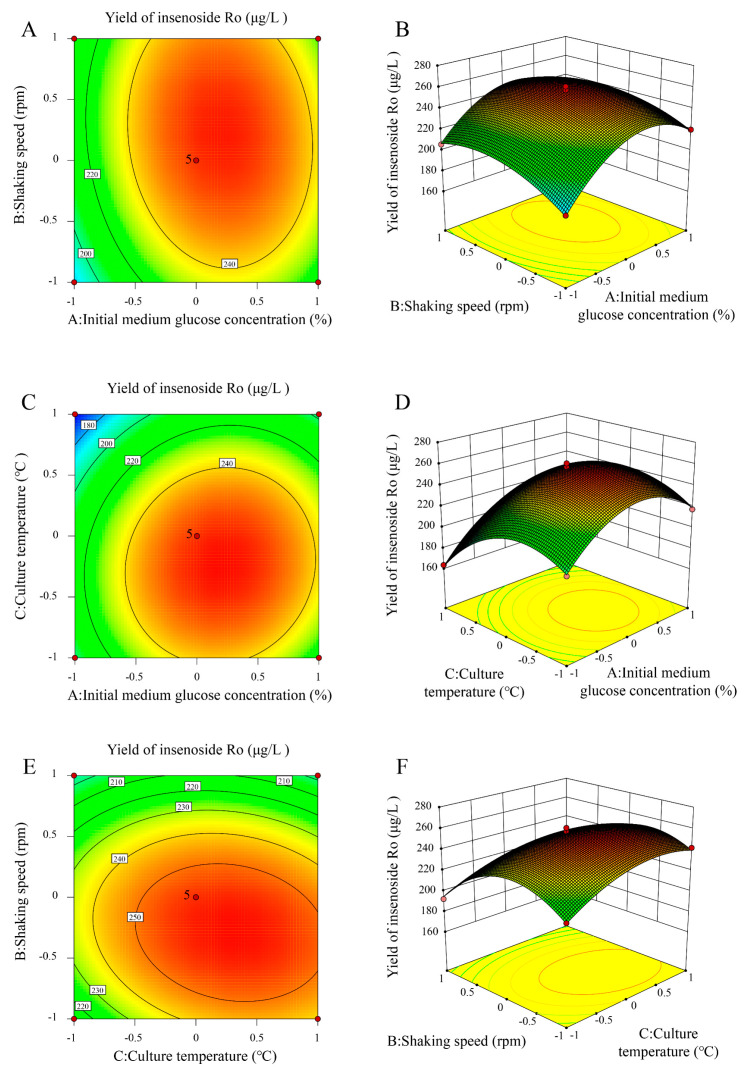
Contour plot and response surface plot of the effect of interaction between factors on the yield of ginsenoside Ro. (**A**,**B**) Shaking speed and initial medium glucose concentration. (**C**,**D**) Culture temperature and initial medium glucose concentration. (**E**,**F**) Shaking speed and culture temperature.

As shown in Figure 3A, when the culture temperature is constant, the contour plot appears as a closed ellipse, and the response surface is convex, indicating a strong interaction between initial medium glucose concentration and shaking speed, with a maximum value. The response surface plot (Figure 3B) shows that with increases in initial medium glucose concentration and shaking speed, the ginsenoside Ro yield initially increases, then decreases. As shown in Figure 3C, when the shaking speed is constant, the contour plot indicates a certain interaction between initial medium glucose concentration and culture temperature, with a maximum value. The response surface plot (Figure 3D) shows that with increases in initial medium glucose concentration and culture temperature, the ginsenoside Ro yield also initially increases, then decreases. As shown in Figure 3E, when the initial medium glucose concentration is constant, the contour plot indicates a certain interaction between shaking speed and culture temperature, with a maximum value. The response surface plot (Figure 3F) shows that with an increase in shaking speed, the ginsenoside Ro yield shows an increasing trend, while with an increase in culture temperature, the ginsenoside Ro yield initially increases, then decreases.

**Figure 4:**
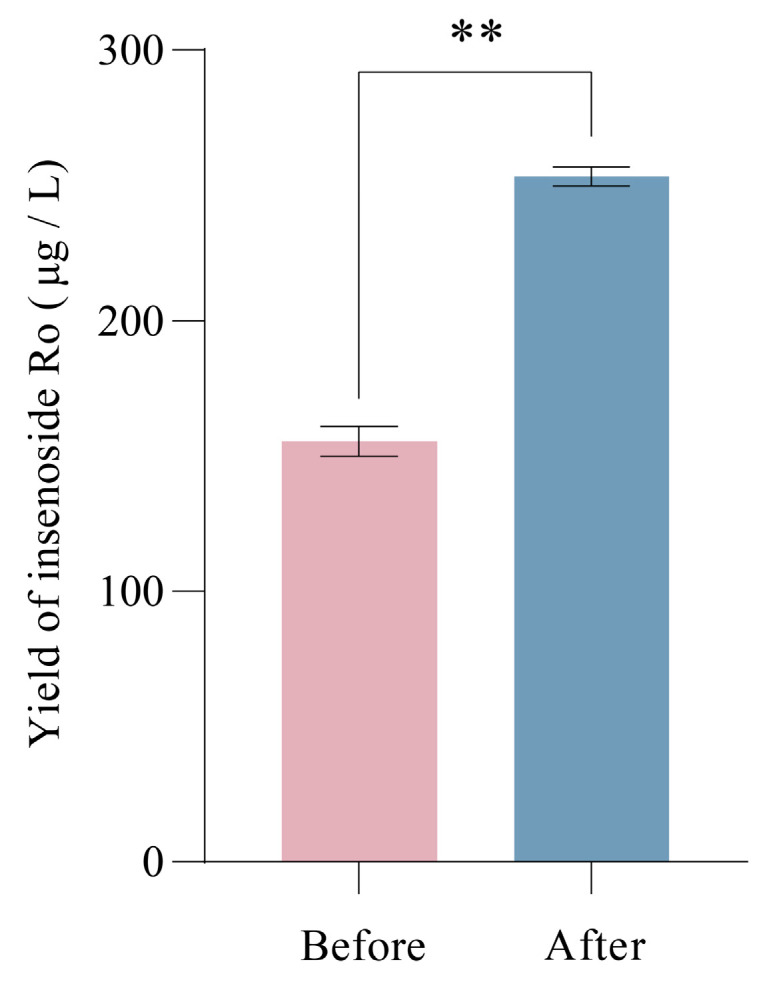
Comparison of the yield of ginsenoside Ro in strain BY-Ro before and after the optimization of fermentation conditions (** for significance of *p* ≤ 0.01).

Based on the above data, the following optimal fermentation conditions are predicted for ginsenoside Ro production by strain BY-Ro: inoculum size, 2%; culture time, 84 h; initial medium glucose concentration, 2.31%; shaking speed, 175.27 rpm; and culture temperature, 31.15 °C. Under these conditions, the predicted ginsenoside Ro yield is 260.19 μg/L. Five validation experiments conducted under approximate levels of the predicted conditions showed an average ginsenoside Ro yield of 253.32 μg/L, with a deviation of 2.64% from the predicted value, indicating good data reproducibility. As shown in Figure 4, the results indicate that the ginsenoside Ro yield after optimization of fermentation conditions is significantly higher than that before optimization, corresponding to 1.63 times the pre-optimization yield.

### 2.5. The Effect of Optimal Fermentation Conditions on the Expression of PgUGAT252645

**Figure 5:**
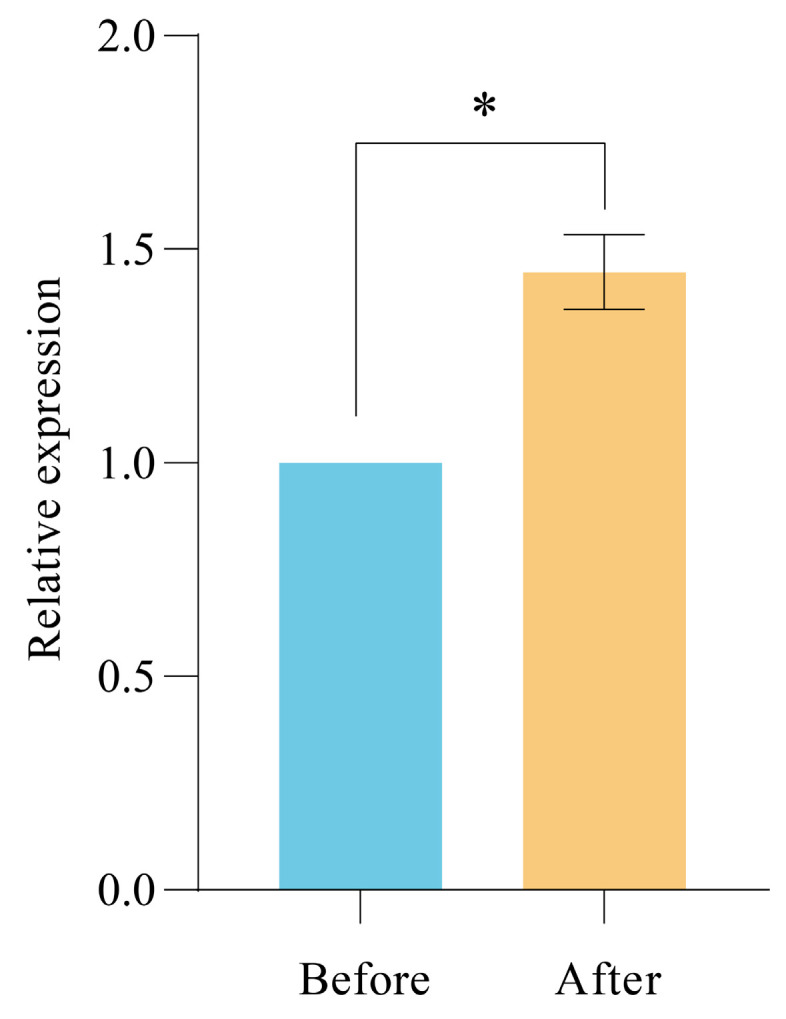
Comparison of the relative expression levels of *PgUGAT252645* in strain BY-Ro before and after the optimization of fermentation conditions (* for significance of *p* ≤ 0.05).

The calculation results of the relative expression of *PgUGAT252645* in strain BY-Ro under different fermentation conditions are shown in Figure 5. The calculation results show that, compared to the strain cultured under pre-optimized conditions, the relative expression of *PgUGAT252645* in strain BY-Ro cultured under optimal fermentation conditions significantly increased to 1.45 times that of the control group. This increase is roughly consistent with the increase in ginsenoside Ro yield, indicating that *PgUGAT252645* is a key enzyme in the constructed heterologous synthesis pathway of ginsenoside Ro, responding to the regulation of culture conditions such as initial medium glucose concentration, culture temperature, and shaking speed.

## 3. Discussion

Synthetic biology research focused on improving product yield often maximizes the optimization of the synthetic pathways for target products at the genetic level. Optimized biosynthetic pathways are often constructed from genes spanning multiple species. For example, after several iterations of updates, some key enzyme genes in the heterologous synthesis pathway of β-carotene have been replaced with modified genes derived from *M. circinelloides* [30]. Although higher yields represent greater application value, such value-driven research may lead to the solidification of synthetic pathway construction schemes. When functional research on new genes is only used for the analysis of synthetic pathways rather than for synthetic applications, the actual value of such genes may be overlooked due to a lack of in-depth exploration.

With the continuous development of relevant technologies, the research focus on heterologous biosynthesis will inevitably expand beyond the key enzyme genes in target pathways. Instead, it will involve more complex studies conducted from a holistic perspective, including investigations of synthesis, metabolism, and regulatory mechanisms. At that time, chassis cells and genetic elements will undergo multidimensional evaluations. The in-depth development of new genes can effectively supplement current genetic resources and prepare for future specific needs. Accordingly, we identified ginsenoside Ro synthesis-related gene *PgUGAT252645* in previous research and used it as one of the key enzyme gene elements to construct ginsenoside Ro biosynthesis-engineered Saccharomyces cerevisiae strain BY-Ro. Preliminary explorations were conducted on the biosynthetic application of *PgUGAT252645*.

*S. cerevisiae* possesses an efficient homologous recombination mechanism based on the DNA double-strand break (DSB) repair model [31]. Only short, homologous arm fragments are needed between the foreign DNA fragments and the genomic DNA of *S. cerevisiae* to trigger homologous recombination, achieving genomic integration of the foreign DNA [32]. Compared to transformation using episomal plasmid vectors, this method provides higher genetic stability and expression efficiency for foreign genes. In this study, we used the pFA6a plasmid series. The target gene fragment was gradually constructed using seamless cloning and other methods and integrated into the *S. cerevisiae* genome. The integration of the target gene and its functional expression were verified by detecting specific products. The advantages of this approach, in addition to having a well-established technical system and highly efficient supporting tools, lie in the high modularity of the gene elements during the assembly of the target gene fragment. This allows for flexible replacement of gene elements to meet different requirements Additionally, since the methods used in this approach are based on fundamental molecular biology techniques, the operating costs can be maintained at a relatively low level.

However, limited integration sites remain a major constraint in constructing engineered *S. cerevisiae* strains using this method. In this study, besides the commonly used rDNA site [33] and δ site [34], we also employed the *tHIS 3* site, which is the residual sequence of the structural *HIS 3* gene present in a single copy in the genome of BY4741, and the *tLEU 2* site, which was artificially constructed and nested within foreign gene fragment C integrated into the *tHIS 3* site. To ensure the integrity of the ginsenoside Ro synthesis pathway, we had to allocate the limited positions to a minimal number of genes. However, product yield and other results indicate that there is still considerable room for optimization in balancing gene copy number and catalytic efficiency under this scheme.

Strain BY-Ro produced 253.32 μg/L of ginsenoside Ro under optimal fermentation conditions. According to subsequent measurements and calculations, this equates to 0.033 mg/g DCW, corresponding to approximately 31% of the ginsenoside Ro content found in plant samples. Although this yield is still significantly lower than the highest reported yield of heterologous biosynthesis of ginsenoside Ro, considering the cost of plant cultivation, including the production cycle, strain BY-Ro still has potential as a production tool for ginsenoside Ro. *PgUGAT252645* sourced from *P. ginseng* also has potential application value as a synthetic biology gene element. However, we have not yet implemented large-scale cultivation of strain BY-Ro in a bioreactor, so we have not achieved the fermentation method commonly used in the heterologous biosynthesis of ginsenosides by *S cerevisiae*, i.e., fed-batch fermentation. Therefore, further research on the fermentation scale and methods is crucial for the enhancement of the production yield of ginsenoside Ro by strain BY-Ro.

The promoters used to construct strain BY-Ro in this study are constitutive promoters known for their ability to maintain relatively stable gene expression under different conditions. However, under the optimized fermentation conditions, the expression level of uridine diphosphate glucose-aldehyde transferase gene *PgUGAT252645* in strain BY-Ro significantly increased. Based on this, we speculate that the introduced exogenous key enzymes have specific requirements for the catalytic environment, such as with respect to temperature, substrate concentration, and oxygen content. These unique conditions may activate regulatory mechanisms within *S cerevisiae*, thereby enhancing transcription levels or the stability of mRNA of downstream genes regulated by relevant promoters. Currently, research on regulatory mechanisms of heterologous biosynthetic systems in *S cerevisiae* is in its early stages. Further in-depth studies in this area could lead to the development of engineered strains with stronger adaptability, aimed at increasing the production yield of target products and further reducing production costs.

## 4. Materials and Methods

### 4.1. Data and Materials

*E. coli* DH5α containing plasmid pET-28a and the chemically competent cells of *E. coli* DH5α were purchased from Sangon Biotech Co., Ltd., Shanghai, China. *S. cerevisiae* S288C direct derivative strain BY4741 [35] was purchased from Huayueyang Biotech Co., Ltd., Beijing, China. The pFA6a-series plasmid vectors listed in Table S1 were purchased from Addgene plasmid repository, Watertown, MA, USA. Codon optimization of open reading frames of key enzyme encoding genes and synthesis of all DNA fragments were completed by Sangon Biotech Co., Ltd., Shanghai, China.

### 4.2. Cloning of Endogenous Gene Elements of BY4741

The BY4741 strain was inoculated into YPD liquid medium and cultured at 30 ℃ and 150 rpm until an OD_600_ of 1.2 was reached. Genomic DNA was then extracted using the TIANamp Yeast DNA Kit (TIANGEN, Beijing, China). Then, using the extracted genomic DNA of BY4741 as the template, endogenous constitutive promoters *TPI1*_P_, *TEF1*_P_, and *TDH3*_P_; terminators *CYC1*_T_, *ADH1*_T_, *FBA1*_T_, and *ENO2*_T_; genomic integration sites; rDNA-locus and δDNA-locus upstream and downstream homologous arms *RU*, *RD*, *DU*, and *DD*; and the C-terminal catalytic domain coding sequence of the rate-limiting enzyme *HMG1* in the MVA pathway, *tHMG1*, were cloned using PrimeSTAR^®^ Max DNA Polymerase (TAKARA, Beijing, China). The sequences of primers, annealing temperatures, and extension times used in this study are listed in Table S2.

### 4.3. Assembly of Key Enzyme Gene Expression Cassettes

Using the Ready-to-Use Seamless Cloning Kit (SANGON, Shanghai, China), the cloned promoters were ligated into the pFA6a-KanMX6 plasmid vector at the *Eco*R I and *Eco*R V restriction sites; the cloned terminators were ligated into the pFA6a-KanMX6 plasmid vector at the *Spe* I and *Sac* II restriction sites; and the cloned *tHMG1* fragments and the exogenous key enzyme genes from different species listed in Table S3, which were codon-optimized for *S. cerevisiae*, were ligated into the pFA6a-KanMX6 plasmid vector at the *Eco*R V and *Spe* I restriction sites to construct the recombinant plasmid vectors shown in Table 8.

During the ligation process, the ligation products were transformed into chemically competent *E. coli* DH5α cells by heat shock and screened on LB solid medium containing ampicillin (final concentration of 100 μg/mL) to obtain positive clones carrying the recombinant plasmid vectors. The positive clones obtained through screening were cultured in LB liquid medium containing ampicillin (final concentration of 100 μg/mL) at 37 °C and 180 rpm until an OD600 of 0.8 was reached. Culturing was then stopped, and the cells were stored at 4 °C for plasmid extraction.

### 4.4. Assembly of Genomic Integration Fragments

Using the Ready-to-Use Seamless Cloning Kit, the cloned upstream homologous *RU* and *DU* arms of the genomic integration sites were ligated into the pET-28a plasmid vector at the *Not* I and *Xho* I restriction sites, and the cloned downstream homologous *RD* and *DD* arms were ligated into the same plasmid vector at the *Bam*H I and *Sac* I restriction sites.

Additionally, residual sequence *tHIS3* of the *HIS3* gene in the BY4741 genome was retrieved from the *Saccharomyces* Genome Database (https://yeastgenome.org/ (accessed on 16 October 2023)) [36], and upstream and downstream homologous *HU* and *HD* arms were designed. Similarly, the sequence of the *LEU2* gene in the *S. cerevisiae* S288C genome was retrieved from the Saccharomyces Genome Database, and a portion of sequence *LEU2* was selected (*tLEU2*) and placed after *HISU* as an additional genomic integration site. Based on the sequence of *tLEU2*, upstream and downstream homologous *LU* and *LD* arms were designed. After the design step, these DNA fragments were synthesized and ligated into the pET-28a plasmid vector at the *Not* I and *Xho* I and *Bam*H I and *Sac* I restriction sites, respectively.

Using recombinant plasmid vectors pK + III, pK + V, and pK + VII as templates, connected key enzyme gene expression cassettes III, V, and VII were cloned. The primer sequences, annealing temperatures, and extension times used in this study are listed in Table S2.

Then, using the Ready-to-Use Seamless Cloning Kit, cloned key enzyme gene expression cassette fragment III was ligated into the pFA6a-5FLAG-hphMX6 plasmid vector at the *Eco*R V and *Spe* I restriction sites. Cloned key enzyme gene expression cassette fragment V was ligated into the pFA6a-URA3 plasmid vector at the *Eco*R I and *Spe* I restriction sites. Cloned key enzyme gene expression cassette fragment VII was ligated into the pFA6a-His3MX6 plasmid vector at the *Eco*R I and *Eco*R V restriction sites, constructing recombinant plasmid vectors for the transfer of selection markers. Subsequently, using these recombinant plasmid vectors and recombinant plasmid vector pK + I as templates, PCR cloning was performed on the combination fragments of the key enzyme gene expression cassettes and selection markers. The primer sequences, annealing temperatures, and extension times are listed in Table S2. The Ready-to-Use Seamless Cloning Kit was used to ligate the cloned combination fragments of the key enzyme gene expression cassettes and selection markers into the *Sac* I and *Sal* I restriction sites of the aforementioned recombinant plasmid vectors containing homologous arm fragments.

Finally, using the constructed recombinant plasmid vectors (pK + II, pK + IV, pK + VI, and pK + VIII) as templates, PCR cloning was performed on the connected key enzyme gene expression cassettes (II, IV, VI, and VIII). The primer sequences, annealing temperatures, and extension times used in this study are listed in Table S2. Using the Ready-to-Use Seamless Cloning Kit, the cloned key enzyme gene expression cassette fragments were ligated into the constructed recombinant plasmid vectors at the *Not* I and *Sal* I restriction sites to create recombinant plasmid vectors with genomic integration fragments for the transformation of *S. cerevisiae*, as shown in Table 9.

### 4.5. Construction of the Engineered S. cerevisiae Strain

To integrate genomic integration fragment A containing the *tHMG1* and beta-amyrin synthase gene (*syn Gg.βAS*) expression cassettes (I, II) into the rDNA site of the genome of BY4741, we used recombinant plasmid vector p28a-A as a template to clone genomic integration fragment A and transformed the BY4741-competent cells using the standard lithium acetate method [37]. The transformed cells were plated on YPDA solid medium containing geneticin G418 (final concentration of 300 μg/mL) for selection to obtain positive strain BY-A.

Subsequently, using recombinant plasmid vector p28a-B as a template, genomic integration fragment B, containing the oleanolic acid synthase gene (*syn Cr.OAS*) and cytochrome P450 reductase gene (*syn Mt.CYPR*) expression cassettes (III, IV), was cloned and transformed into BY-A-competent cells, using the aforementioned method to integrate it into the δDNA site of the genome of BY-A. Then, strain BY-OA was obtained by selection on YPDA solid medium containing geneticin G418 (final concentration of 300 μg/mL) and hygromycin B (final concentration of 500 μg/mL).

The construction of strain BY-CE was achieved by using the aforementioned method to integrate genomic integration fragment C, which contains the uridine diphosphate glucose dehydrogenase gene (*syn At.UGDH*) and the uridine diphosphate glucuronic acid transferase gene (*syn Pg.UGAT*) expression cassette, into the *HIS3* residual sequence site of the genome of BY-OA. Strain BY-CE was obtained by selection on uracil-deficient SD solid medium containing geneticin G418 (final concentration of 300 μg/mL) and hygromycin B (final concentration of 500 μg/mL).

Strain BY-RO was constructed by using the aforementioned method to integrate genomic integration fragment D, which contains two uridine diphosphate glucose transferase gene (*Syn Mt.UGT* and *Syn Ts.UGT*) expression cassettes, into the artificially added *tLEU* sequence site of the genome of BY-CE. Strain BY-Ro was screened on SD solid medium deficient in uracil and histidine containing geneticin G418 (final concentration of 300 μg/mL) and hygromycin B (final concentration of 500 μg/mL).

### 4.6. Detection of Target Products

During the strain construction process, all screened positive strains, except for strain BY-A, were inoculated into YPD liquid medium and cultured at 30 ℃ and 150 rpm until an OD600 of 1.2 was reached. The cells were then disrupted using an ultrasonic cell disruptor. The cell lysate was then centrifuged at room temperature and 12,000 rpm for 45 min to collect the supernatant. The filtered supernatant was then loaded onto a separation column packed with octadecylsilane (ODS)-bonded silica gel. After the supernatant completely passed through the column, impurities were eluted with 5 column volumes of 40% methanol solution. Finally, the separated products were eluted with 5 column volumes of 100% methanol, the eluate was collected and evaporated to dryness, and the residue was dissolved in chromatographic methanol. The target products of each strain were then detected using Agilent 1290-6470B LC-MS/MS (Agilent, Santa Clara, CA, USA) under the conditions described in Table S4.

### 4.7. Single-Factor Experiment

Using 500 mL Erlenmeyer flasks with 250 mL standard YPD liquid medium, an inoculum size of 3%, a shaking speed of 150 rpm, a culture temperature of 30 ℃, and a culture time of 84 h as the initial culture conditions, the effects of five major factors on the yield of ginsenoside Ro in strain BY-RO were studied in sequence as follows: inoculum size, 1%, 2%, 3%, 4%, and 5%; culture time, 60 h, 72 h, 84 h, 96 h, and 108 h; shaking speed, 110 rpm, 130 rpm, 150 rpm, 170 rpm, and 190 rpm; culture temperature, 26 °C, 28 °C, 30 °C, 32 °C, and 34 °C; and initial medium glucose concentration, 1%, 1.5%, 2%, 2.5%, and 3%. Three biological replicates were set up for each condition. After cultivation, the ginsenoside Ro in the cultures was extracted and detected using the aforementioned method.

### 4.8. Experiment and Analysis of the Plackett–Burman Design

By using the Plackett–Burman Design function in Design-Expert (Version 10.0.3), the Plackett–Burman experiment was designed based on the results of the aforementioned single-factor experiments and conducted according to the designed protocol. Subsequently, through statistical analysis of the results obtained from the Plackett-Burman experiment, factors significantly affecting the yield of ginsenoside Ro in strain BY-Ro were selected among inoculum size, culture time, shaking speed culture temperature, and initial medium glucose concentration to obtain fundamental data for subsequent response surface optimization experiments.

### 4.9. Experiment and Analysis of the Box—Behnken Design

Using the Box–Behnken Design function in Design-Expert, the response surface methodology was employed to design optimization experiments based on the results of the Plackett–Burman experiment, with subsequent experiments conducted accordingly. Ginsenoside Ro yield was used as the response value, with three levels set for each factor. Subsequently, the fermentation conditions for strain BY-Ro were optimized based on the statistical analysis of the experimental optimization results. Finally, verification experiments were conducted under the optimal fermentation conditions to validate the effectiveness of the established model.

### 4.10. The Effect of Optimal Fermentation Conditions on the Expression of PgUGAT252645

After fermenting strain BY-Ro under optimal fermentation conditions, total RNA was extracted from strain BY-Ro using TRNzol Universal total RNA extraction reagent (TIANGEN, Beijing, China). A PrimeScript™ FAST RT reagent Kit with gDNA Eraser (TAKARA, Beijing, China) was then used to further remove genomic DNA from the RNA samples, and the RNA was reverse-transcribed into cDNA. Subsequently, TB Green^®^ Fast qPCR Mix (TAKARA, Beijing, China) was used with the primers listed in Table S2, using cDNA as the template and *S. cerevisiae* TATA-binding protein-associated factor gene *TAF10* [38] as the reference gene. Real-time quantitative PCR was performed using an Applied Biosystems 7500 Real-Time PCR System. Strain BY-Ro cells cultured under pre-optimization fermentation conditions were used as controls to detect changes in the expression levels of the key enzyme gene *PgUGAT252645* before and after optimization of fermentation conditions.

## 5. Conclusions

Using *PgUGAT252645* as identified in previous studies and several other reported key enzyme genes, as well as the endogenous promoters and terminators of *S. cerevisiae* BY4741, as elements, various plasmid vectors were employed to assemble genomic integration fragments. These genomic integration fragments were transferred into chemically competent *S. cerevisiae* cells in batches, successfully constructing strain BY-Ro for the biosynthesis of ginsenoside Ro. Among various fermentation conditions, the culture temperature, initial medium glucose concentration, and shaking speed significantly impacted the ginsenoside Ro yield in strain BY-Ro. The interaction between shaking speed and culture temperature also significantly affected the ginsenoside Ro yield. Under the initially optimized fermentation conditions, the ginsenoside Ro yield in strain BY-Ro was 253.32 μg/L, which is 1.63 times the yield before optimization. *PgUGAT252645*, as a key enzyme gene, responded to the regulation of culture conditions, with its relative expression level increasing to 1.45 times that before the optimization of fermentation conditions. This study not only deepened the understanding of the function of *PgUGAT252645* but also explored its potential application as a genetic element for the industrial production of ginsenoside Ro.

## Figures and Tables

**Table 1:** Information on engineered brewing yeast strains.

Name	Description
BY4741	*MATα his3Δ1 leu2Δ0 met15Δ0 ura3Δ0*
BY-A	*BY4741*, *rDNA:: TPI 1P-tHMG 1-CYC 1T-KanR-TEF 1P-syn Gg.βAS-ADH 1T*
BY-OA	*BY-A*, *δDNA:: TEF 1P-syn Cr.OAS-FBA 1T-HygR-TDH 3P-syn Mt.CYPR-ENO 2T*
BY-CE	*BY-OA*, *tHIS 3:: TPI 1P-syn At.UGDH-CYC 1T-URA 3-TEF 1P-syn Pg.UGAT-ADH 1T*
BY-Ro	*BY-CE*, *tLEU 2:: TEF 1P-syn Mt.UGT-FBA 1T-HIS 3-TDH 3P-syn Ts.UGT-ENO 2T*

**Table 2:** Factors and levels of the Plackett–Burman design.

Factor	Levels	
	−1	1
A. Inoculum Size (%)	2	4
B. Culture Time (h)	60	84
C. Shaker Speed (rpm)	150	190
D. Culture Temperature (°C)	30	34
E. Medium Glucose Concentration (%)	2	3

**Table 3:** Plackett–Burman design and experimental results.

Experiment Number	Factors					Ginsenoside Ro Yield (μg/L)
	A	B	C	D	E	
1	−1	1	1	−1	−1	231.11
2	−1	1	1	1	1	166.88
3	1	−1	1	−1	1	214.16
4	−1	−1	−1	1	−1	145.55
5	1	1	−1	1	1	171.51
6	1	1	1	−1	−1	224.48
7	−1	−1	−1	−1	−1	199.32
8	−1	−1	1	1	1	145.55
9	1	−1	1	1	−1	154.82
10	−1	1	−1	−1	1	221.35
11	1	−1	−1	−1	1	207.67
12	1	1	−1	1	−1	176.16

**Table 4:** Statistical analysis of the Plackett–Burman design and experimental results.

Source of Variation	Sum of Squares	Degrees of Freedom	Mean Square	F Value	*p* Value	Significance
Model	10,937.83	5	2187.57	104.22	<0.0001	**
A	1.56	1	1.56	0.074	0.7945	
B	19.89	1	19.89	0.95	0.368	
C	126.82	1	126.82	6.04	0.0493	*
D	9499.83	1	9499.83	452.58	<0.0001	**
E	1289.75	1	1289.75	61.44	0.0002	**
Residual	125.94	6	20.99			
Total	11,063.78	11				

* for significance of *p* ≤ 0.05 and ** for significance of *p* ≤ 0.01.

**Table 5:** Factors and levels of the Box–Behnken design.

Factor	Levels		
	−1	0	1
A. Medium Glucose Concentration (%)	2	2.5	3
B. Shaker Speed (rpm)	150	170	190
C. Culture Temperature (℃)	30	32	34

**Table 6:** Box–Behnken design and experimental results.

Experiment Number	Factors			Ginsenoside Ro Yield (μg/L)
	A	B	C	
1	0	0	0	257.44
2	−1	0	1	163.52
3	0	1	−1	242.06
4	−1	0	−1	200.13
5	0	0	0	254.92
6	0	−1	1	192.59
7	0	1	1	196.50
8	1	0	−1	217.74
9	0	−1	−1	214.11
10	1	1	0	224.73
11	0	0	0	253.52
12	0	0	0	253.24
13	−1	1	0	206.00
14	0	0	0	260.79
15	1	0	1	198.46
16	−1	−1	0	185.60
17	1	−1	0	220.26

**Table 7:** Statistical analysis of the Box–Behnken design and experimental results.

Source of Variation	Sum of Squares	Degrees of Freedom	Mean Square	F Value	*p* Value	Significance
Model	13,732.47	9	1525.83	173.03	<0.0001	**
A	1402.84	1	1402.84	159.09	<0.0001	**
B	402.46	1	402.46	45.64	0.0003	**
C	1890.75	1	1890.75	214.42	<0.0001	**
AB	63.46	1	63.46	7.2	0.0314	*
AC	75.08	1	75.08	8.51	0.0224	*
BC	144.46	1	144.46	16.38	0.0049	**
A^2^	4202.46	1	4202.46	476.57	<0.0001	**
B^2^	978.02	1	978.02	110.91	<0.0001	**
C^2^	3645.91	1	3645.91	413.46	<0.0001	**
Lack of fit	21.82	3	7.27	0.73	0.5861	
Residual	61.73	7	8.82			
Pure error	39.91	4	9.98			
Total	13,794.2	16				

* for significance of *p* ≤ 0.05 and ** for significance of *p* ≤ 0.01.

**Table 8:** Recombinant plasmid vectors containing the key enzyme gene expression cassette.

Name	Promoter	Key Enzyme Gene	Terminator
pK + I	*TPI 1P*	*tHMG 1*	*CYC 1T*
pK + II	*TEF 1P*	*Syn Gg.βAS*	*ADH 1T*
pK + III	*TEF 1P*	*Syn Cr.OAS*	*FBA 1T*
pK + IV	*TDH 3P*	*Syn Mt.CYPR*	*ENO 2T*
pK + V	*TPI 1P*	*Syn At.UGDH*	*CYC 1T*
pK + VI	*TEF 1P*	*Syn Pg.UGAT*	*ADH 1T*
pK + VII	*TEF 1P*	*Syn Mt.UGT*	*FBA 1T*
pK + VIII	*TDH 3P*	*Syn Ts.UGT*	*ENO 2T*

**Table 9:** Recombinant plasmid vector containing integrated genomic integration genetic transformation fragments.

Name	Upstream Homologous Arm	Expression Cassette 1	Selection Marker	Expression Cassette 2	Downstream Homologous Arm
p28a-A	*RU*	I	*KanR*	II	*RD*
p28a-B	*DU*	III	*HygR*	IV	*DD*
p28a-C	*HU + tLEU 2*	V	*URA 3*	VI	*HD*
p28a-D	*LU*	VII	*HIS 3*	VIII	*LD*

## Data Availability

Data are contained within the article.

## Supplementary Materials

The following supporting information can be downloaded at: https://www.mdpi.com/article/10.3390/ijms252011331/s1.

## References

[1] Wei G., Yang F., Wei F., Zhang L., Gao Y., Qian J., Chen Z., Jia Z., Wang Y., Su H. (2020). Metabolomes and transcriptomes revealed the saponin distribution in root tissues of *Panax quinquefolius* and *Panax notoginseng*. J. Ginseng Res..

[2] Liang Y., Zhao S. (2008). Progress in understanding of ginsenoside biosynthesis. Plant Biol..

[3] Kim Y.J., Zhang D., Yang D.C. (2015). Biosynthesis and biotechnological production of ginsenosides. Biotechnol. Adv..

[4] Yang J.L., Hu Z.F., Zhang T.T., Gu A.D., Gong T., Zhu P. (2018). Progress on the Studies of the Key Enzymes of Ginsenoside Biosynthesis. Molecules.

[5] Han J.Y., Kim H.J., Kwon Y.S., Choi Y.E. (2011). The Cyt P450 Enzyme CYP716A47 Catalyzes the Formation of Protopanaxadiol from Dammarenediol-II During Ginsenoside Biosynthesis in *Panax ginseng*. Plant Cell Physiol..

[6] Wu W., Lu Z., Teng Y., Guo Y., Liu S. (2016). Structural Characterization of Ginsenosides from Flower Buds of *Panax ginseng* by RRLC-Q-TOF MS. J. Chromatogr. Sci..

[7] Kim J.S. (2022). Optimization of Accelerated Solvent Extraction of Ginsenosides from Cultivated Wild Ginseng Using Response Surface *Methodology*. Prev. Nutr. Food Sci..

[8] Roberts S.C. (2007). Production and engineering of terpenoids in plant cell culture. Nat. Chem. Biol..

[9] Murthy H.N., Dandin V.S., Paek K.Y. (2016). Tools for biotechnological production of useful phytochemicals from adventitious root cultures. Phytochem. Rev..

[10] Choi H.S., Koo H.B., Jeon S.W., Han J.Y., Kim J.S., Jun K.M., Choi Y.E. (2022). Modification of ginsenoside saponin composition via the CRISPR/Cas9-mediated knockout of protopanaxadiol 6-hydroxylase gene in *Panax ginseng*. J. Ginseng Res..

[11] Dai Z., Liu Y., Zhang X., Shi M., Wang B., Wang D., Huang L., Zhang X. (2013). Metabolic engineering of Saccharomyces cerevisiae for production of ginsenosides. Metab. Eng..

[12] Li D., Zhang Q., Zhou Z., Zhao F., Lu W. (2016). Heterologous biosynthesis of triterpenoid dammarenediol-II in engineered *Escherichia coli*. Biotechnol. Lett..

[13] Gwak Y.S., Han J.Y., Adhikari P.B., Ahn C.H., Choi Y.E. (2017). Heterologous production of a ginsenoside saponin (compound K) and its precursors in transgenic tobacco impairs the vegetative and reproductive growth. Planta.

[14] Gwak Y.S., Han J.Y., Choi Y.E. (2019). Production of ginsenoside aglycone (protopanaxatriol) and male sterility of transgenic tobacco co-overexpressing three *Panax ginseng* genes: PgDDS, CYP716A47, and CYP716A53v2. J. Ginseng Res..

[15] Chen Q., Liu D., Qu Y., Lei J., Zhang J., Cui X., Ge F. (2023). Construction of plant cell factory for biosynthesis of ginsenoside Rh2 in tobacco. Ind. Crops Prod..

[16] Pompon D., Louerat B., Bronine A., Urban P. (1996). Yeast expression of animal and plant P450s in optimized redox environments. Methods Enzymol..

[17] Ren H., Hu P., Zhao H. (2017). A plug-and-play pathway refactoring workflow for natural product research in *Escherichia coli* and *Saccharomyces cerevisiae*. Biotechnol. Bioeng..

[18] Wang C.L., Liwei M., Park J.B., Jeong S.H., Wei G.Y., Wang Y.J., Kim S.W. (2018). Microbial Platform for Terpenoid Production: *Escherichia coli* and Yeast. Front. Microbiol..

[19] Wang D., Wang J., Shi Y., Li R., Fan F., Huang Y., Li W., Chen N., Huang L., Dai Z. (2020). Elucidation of the complete biosynthetic pathway of the main triterpene glycosylation products of Panax notoginseng using a synthetic biology platform. Metab. Eng..

[20] Dai Z., Wang B., Liu Y., Shi M., Wang D., Zhang X., Liu T., Huang L., Zhang X. (2014). Producing aglycons of ginsenosides in bakers’ yeast. Sci. Rep..

[21] Yan X., Fan Y., Wei W., Wang P., Liu Q., Wei Y., Zhang L., Zhao G., Yue J., Zhou Z. (2014). Production of bioactive ginsenoside compound K in metabolically engineered yeast. Cell Res..

[22] Ren S., Sun Q., Zhang L., Sun W., Li Y., Feng X., Li C. (2022). Sustainable production of rare oleanane-type ginsenoside Ro with an artificial glycosylation pathway in *Saccharomyces cerevisiae*. Green Chem..

[23] Zhang H., Hua X., Zheng D., Wu H., Li C., Rao P., Wen M., Choi Y.E., Xue Z., Wang Y. (2022). De Novo Biosynthesis of Oleanane-Type Ginsenosides in *Saccharomyces cerevisiae* Using Two Types of Glycosyltransferases from *Panax ginseng*. J. Agric. Food Chem..

[24] Shi Y., Wang D., Li R., Huang L., Dai Z., Zhang X. (2021). Engineering yeast subcellular compartments for increased production of the lipophilic natural products ginsenosides. Metab. Eng..

[25] Wang P., Wei W., Ye W., Li X., Zhao W., Yang C., Li C., Yan X., Zhou Z. (2019). Synthesizing ginsenoside Rh2 in *Saccharomyces cerevisiae* cell factory at high-efficiency. Cell Discov..

[26] Zhao C., Gao X., Liu X., Wang Y., Yang S., Wang F., Ren Y. (2016). Enhancing Biosynthesis of a Ginsenoside Precursor by Self-Assembly of Two Key Enzymes in Pichia pastoris. J. Agric. Food Chem..

[27] Wu Y., Xu S., Gao X., Li M., Li D., Lu W. (2019). Enhanced protopanaxadiol production from xylose by engineered Yarrowia lipolytica. Microb. Cell Factories.

[28] Wang Y., Zhang H., Ri H.C., An Z., Wang X., Zhou J.-N., Zheng D., Wu H., Wang P., Yang J. (2022). Deletion and tandem duplications of biosynthetic genes drive the diversity of triterpenoids in *Aralia elata*. Nat. Commun..

[29] Yu X., Yu J., Liu S., Liu M., Wang K., Zhao M., Wang Y., Chen P., Lei J., Wang Y. (2024). Transcriptome-Wide Identification and Integrated Analysis of a UGT Gene Involved in Ginsenoside Ro Biosynthesis in *Panax ginseng*. Plants.

[30] Varghese R., Buragohain T., Banerjee I., Mukherjee R., Penshanwar S.N., Agasti S., Ramamoorthy S. (2023). The apocarotenoid production in microbial biofactories: An overview. J. Biotechnol..

[31] Lettier G., Feng Q., de Mayolo A.A., Erdeniz N., Reid R.J.D., Lisby M., Mortensen U.H., Rothstein R. (2006). The role of DNA double-strand breaks in spontaneous homologous recombination in S. cerevisiae. PLoS Genet..

[32] Yoshida J., Umezu K., Maki H. (2003). Positive and negative roles of homologous recombination in the maintenance of genome stability in *Saccharomyces cerevisiae*. Genetics.

[33] Zheng H., Wang K., Xu X., Pan J., Sun X., Hou J., Liu W., Shen Y. (2022). Highly efficient rDNA-mediated multicopy integration based on the dynamic balance of rDNA in *Saccharomyces cerevisiae*. Microb. Biotechnol..

[34] Yamada R., Tanaka T., Ogino C., Fukuda H., Kondo A. (2010). Novel strategy for yeast construction using δ-integration and cell fusion to efficiently produce ethanol from raw starch. Appl. Microbiol. Biotechnol..

[35] Winston F., Dollard C., Ricupero-Hovasse S.L. (1995). Construction of a set of convenient *Saccharomyces cerevisiae* strains that are isogenic to S288C. Yeast.

[36] Wong E.D., Miyasato S.R., Aleksander S., Karra K., Nash R.S., Skrzypek M.S., Weng S., Engel S.R., Cherry J.M. (2023). Saccharomyces genome database update: Server architecture, pan-genome nomenclature, and external resources. Genetics.

[37] Amberg D.C., Burke D.J., Strathern J.N., David C. (2005). Methods in Yeast Genetics: A Cold Spring Harbor Laboratory Course Manual.

[38] Teste M.A., Duquenne M., Francois J.M., Parrou J.L. (2009). Validation of reference genes for quantitative expression analysis by real-time RT-PCR in *Saccharomyces cerevisiae*. BMC Mol. Biol..

